# Josephson effects in the junction formed by *DIII*-class topological and *s*-wave superconductors with an embedded quantum dot

**DOI:** 10.1038/srep28311

**Published:** 2016-06-21

**Authors:** Zhen Gao, Xiao-Qi Wang, Wan-Fei Shan, Hai-Na Wu, Wei-Jiang Gong

**Affiliations:** 1 College of Sciences, Northeastern University, Shenyang 110819, China

## Abstract

We investigate the Josephson effects in the junction formed by the indirect coupling between *DIII*-class topological and *s*-wave superconductors via an embedded quantum dot. Due to the presence of two kinds of superconductors, three dot-superconductor coupling manners are considered, respectively. As a result, the Josephson current is found to oscillate in period 2*π*. More importantly, the presence of Majorana doublet in the *DIII*-class superconductor renders the current finite at the case of zero phase difference, with its sign determined by the fermion parity of such a junction. In addition, the dot-superconductor coupling plays a nontrivial role in adjusting the Josephson current. When the *s*-wave superconductor couples to the dot in the weak limit, the current direction will have an opportunity to reverse. It is believed that these results will be helpful for understanding the transport properties of the *DIII*-class superconductor.

Topological superconductors (TSs) have received much attention from experimental and theoretical aspects because Majorana modes appear at the ends of the one-dimensional TS which can potentially be used for topological quantum computation^1^,^2^,^3^,^4^. Due to the possibility of achieving Majorana modes, the systems with TSs show abundant and interesting physical characteristics^5^,^6^. For instance, in the proximity-coupled semiconductor-TS devices, the Majorana zero modes induce the zero-bias anomaly^7^,^8^. A more compelling TS signature is the unusual Josephson current-phase relation. Namely, when the normal s-wave superconductor nano-wire is replaced by a TS wire with Majorana zero modes, the current-phase relation will be modified to be 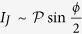 with the period 4*π* (*ϕ* is the superconducting phase difference and 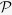 is the fermion parity). This is the so-called the fractional Josephson effect^9^,^10^,^11^,^12^,^13^,^14^,^15^.

More recently, the time-reversal invariant TSs, i.e., the *DIII* symmetry-class TSs^16^,^17^,^18^,^19^,^20^, have become one new concern^21^,^22^,^23^. In such a kind of TSs, the zero modes appear in pairs due to Kramers’s theorem, differently from the chiral TSs. As a result, two Majorana bound states (MBSs) will be localized at each end of the *DIII*-TS nanowire, leading to the formation of one Kramers doublet^24^,^25^. Since the Kramers doublet is protected by the time-reversal symmetry, it will inevitably drive some new transport phenomena, opposite to the single Majorana zero mode. Up to now, many groups have proposed proposals to achieve the *DIII*-TS nanowires, by using the proximity effects of *d*-wave, *p*-wave, *s*±-wave, or conventional *s*-wave superconductors^26^,^27^,^28^,^29^,^30^,^31^. Meanwhile, physicists have dedicated themselves to the quantum transport phenomena contributed by Majorana doublet, and some important results have been reported^32^,^33^,^34^. For instance, in the Josephson junction formed by the *DIII*-class TSs with Majorana doublets, the period of the Josephson current will be varied by the change of fermion parity (FP) in this system^33^. This exactly means the interesting and nontrivial role of Majorana doublet in contributing to the Josephson effect. However, it should be noticed that to further understand the physical property of Majorana doublet, Josephson effects in any new junctions should be investigated.

Due to the presence of Majorana doublet, finite Josephson current can be driven by the Josephson phase difference when the *DIII*-class TS couples to one *s*-wave superconductor. This phenomenon is basically different from the *D*-class TS. Following this idea, we would like to investigate the Josephson effect in such a topological/nontopological junction. Also, for presenting the detailed current property, we embed one quantum dot (QD) between the superconductors with the reason as follows. QD is a typical mesoscopic cell characterized by its discrete level and strong Coulomb interaction, which play an important role in influencing the Josephson effect. Besides, QD is able to accommodate an electron and the average electron occupation in one QD can be changed by shifting the QD level. Thus, when one QD is introduced in the TS junction, FP can be re-regulated and then the Josephson current can be modified as well. It is therefore anticipated that interesting Josephson effect can be induced by the interplay among the Majorana doublet, the regular bound states in the QD, and the Cooper pair in the *S*-wave superconductor. Our calculations show that although the Josephson current oscillates in 2*π* period, the presence of Majorana doublet in the *DIII*-class TS renders it finite at the case of zero phase difference, with its sign determined by the fermion parity of the whole system. Besides, QD modulates the Josephson effect in a nontrivial way. In the extreme case where the *s*-wave superconductor couples weakly to the QD, the direction of the Josephson current will be reversed. All these results describe the specific role of *DIII*-class TS in driving the Josephson effect.

## Theoretical Model

**Figure 1:**
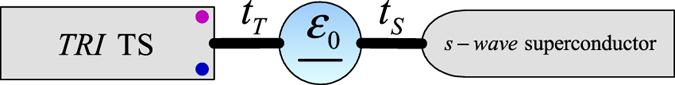
The junction formed by *DIII*-class topological and *s*-wave superconductors. One QD is embedded in the junction.

The considered Josephson junction is illustrated in Fig. 1, where a one-dimensional *DIII*-class TS couples to one *s*-wave superconductor via one QD. The Hamiltonian of such a system can be written as *H* = *H*_*p*_ + *H*_*s*_ + *H*_*D*_ + *H*_*T*_. The first two terms, i.e., *H*_*p*_ and *H*_*s*_, denote the Hamiltonians of the *DIII*-class TS and s-wave superconductor respectively, which is written as^32^
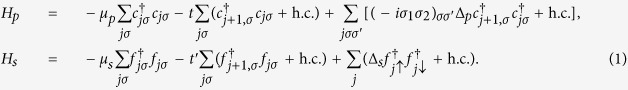

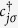
 and 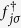 (*c*_*jσ*_ and *f*_*jσ*_) are the electron creation (annihilation) operators in the *DIII*-class TS and *s*-wave superconductor, respectively, with *σ* being the spin index. *μ*_*p*_ and *μ*_*s*_ are the chemical potentials of the superconductors, and Δ_*p*_ and Δ_*s*_ are the Copper-pair hopping terms. *H*_*D*_ describes the Hamiltonian of the QD. For a single-level QD, it takes the form as *H*_*D*_ = *H*_*d*0_ + *H*_*ee*_ with
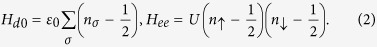

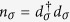
 is the electron-number operator, in which 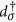 and *d*_*σ*_ are the creation and annihilation operators in the QD. *ε*_0_ is the QD level, and *U* denotes the intradot Coulomb repulsion. *H*_*T*_, the last term of *H*, represents the couplings between the QD and the superconductors. It can be given by
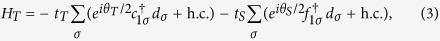
where *t*_*T*_ and *t*_*S*_ are the QD-superconductor coupling coefficients, respectively.

It is well-known that the phase difference between superconductors drives finite current through one Josephson junction. With respect to such a junction, the current properties can be evaluated by the following formula
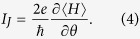
*θ* = *θ*_*T*_ − *θ*_*S*_ is the phase difference between the superconductors, and 〈···〉 is the thermal average. As a typical case, i.e., the zero-temperature limit, the Josephson current can be simplified as
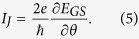


This formula indicates that the calculation about the Josephson current is dependent on the solution of the ground-state (GS) level of this system.

In the low-energy region, the *DIII*-class TS only contributes Majorana doublets to the Josephson effect, so *H*_*p*_ describes the coupling between two Majorana doublets. For the extreme case of one infinitely-long TS, the coupling strength between the Majorana doublets decreases to zero^35^. Following this idea, we project *H*_*T*_ onto its zero-energy subspace. As a result, *H* can be rewritten as
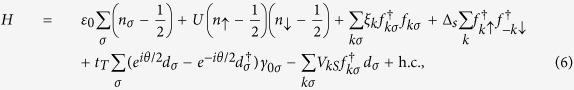
where *H*_*s*_ has been projected into in the Bloch space with *ξ*_*k*_ = −*μ*′ − 2*t*′cos*k* and 
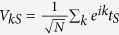
. *γ*_0*σ*_, the Majorana operator, which obeys the anti-commutation relationship of {*γ*_0*σ*_, *γ*_0*σ*′_} = 2*δ*_*σσ*′_. Based on the renewed expression of *H*, we next try to diagonalize the Hamiltonian of such a Josephson junction.

## Diagonalization of the Junction Hamiltonian

The continuum state in the *s*-wave superconductor hinders the diagonalization of the system’s Hamiltonian. In order to present a comprehensive analysis, we would like to consider three cases, i.e., the cases of *t*_*T*_ ≫ *t*_*S*_, *t*_*T*_ ≈ *t*_*S*_, and *t*_*T*_ ≪ *t*_*S*_, followed by the application of different approximation methods. The following are the detailed discussion processes. For convenience, they are named as Case I, Case II, and Case III, respectively.

## Diagonalization of *H* in Case I

In Case I where *t*_*T*_ ≫ *t*_*S*_, the subsystem formed by the QD and Majorana doublet can be considered to be one system, whereas the *s*-wave superconductor can be viewed as a perturbation factor. We next simplify the system Hamiltonian with the help of the perturbation theory. Ignoring the Coulomb interaction term in the QD, we can write out the action of the subsystem of QD and *s*-wave superconductor
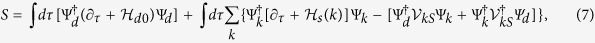
where 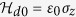, 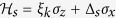, and 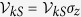 with *σ*_*α*_ being the pauli matrix (*σ* = *x*, *y*, *z*). As for the field operators, they are given by 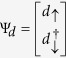 and 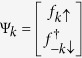. With the action *S*, we can express the partition function as a path integral, i.e., 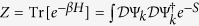, in which the measure 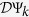 denotes all the possible integral paths. After integrating out the fermion field 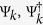 with a Gaussian integral, the partition function will become a “generating functional”
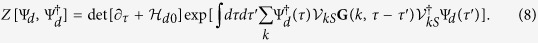

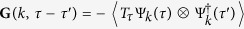
 is defined in the *s*-wave superconductor. It obeys the Fourier expansion 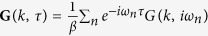 with 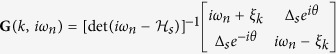. This allows us to write out the effective expression of the action in the Fourier space directly, i.e.,
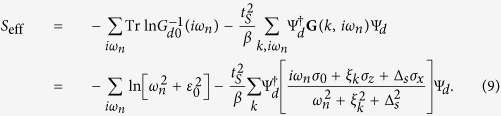


According to the expression of *S*_eff_, it is not difficult for us to get the perturbative Hamiltonian of the *s*-wave superconductor on the QD
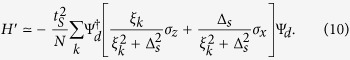


Via a unitary transformation, the system Hamiltonian can be expressed as the following form
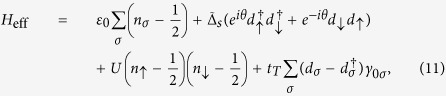
with 
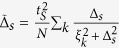
. Such a result indicates that the weak Andreev reflection between the *s*-wave superconductor and QD induces a weak *s*-wave pairing potential on the QD, which is exactly the so-called the proximity effect.

For the sake of diagonalizing such a Hamiltonian, we need to introduce local Majorana operators *η*_*iσ*_ through 
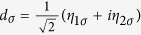
 and 
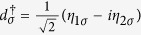
. And then, by defining Dirac fermionic operators 
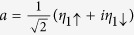
, 
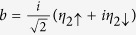
, and 
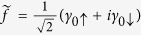
, we can obtain a new expression of *H*_eff_, i.e.,
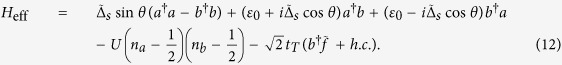


Based on Eq. (12), the Bogoliubov-de Gennes equation *H*_eff_Ψ = *E*Ψ can be built up, and then the eigenvalues of *H*_eff_ can be worked out. On the basis of {|000〉, |001〉, |010〉, |100〉, |110〉, |101〉, |011〉, |111〉}, the matrix form of *H* can be obtained (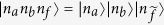, where *n*_*a*_ = *a*^†^*a*, *n*_*b*_ = *b*^†^*b*, and 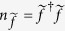). Note that in the TS-existed system, only the parity of the average particle occupation number is the good quantum number, thus the matrix form of *H*_eff_ should be given according to FP. In the case of odd FP, Ψ_*o*_ = *c*_1_|001〉 + *c*_2_|010〉 + *c*_3_|100〉 + *c*_4_|111〉, and then
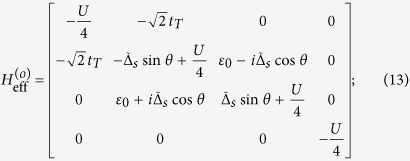


In the case of even FP, Ψ_*e*_ = *c*_1_|000〉 + *c*_2_|011〉 + *c*_3_|101〉 + *c*_4_|110〉, and
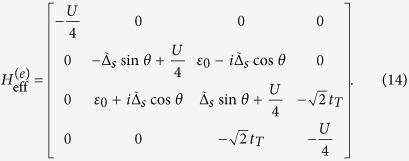


It is easy to find that 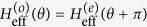. Thus, the Josephson effect can be clarified by paying attention to the current oscillation result in one FP.

## Diagonalization of *H* in Case II

In Case II where *t*_*T*_ ≈ *t*_*S*_, *H* is difficult to diagonalize due to the presence of continuum state in the *s*-wave superconductor. However, according to the previous works, the zero band-width approximation is feasible to solve the Josephson effect contributed by the *s*-wave superconductor^36^. Within such an approximation, the Hamiltonian can be simplified as
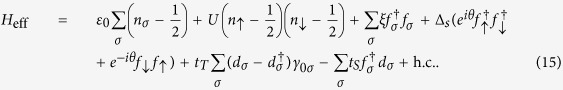


By defining 
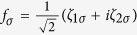
 and 
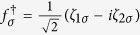
 with 
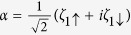
 and 
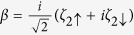
, we get the Hamiltonian in the spinless-fermion representation
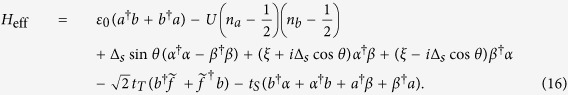


On the basis of odd FP, the matrix of 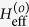 can be expressed as
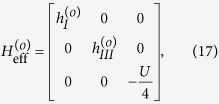
where
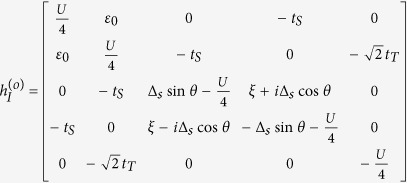
and
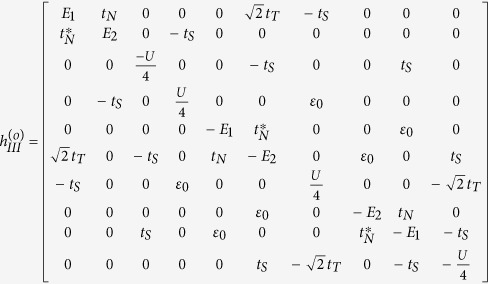
with 
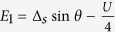
, 
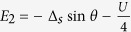
, and *t*_*N*_ = *ξ* + *i*Δ_*s*_cos*θ*. On the basis of even FP, the matrix of 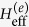 can be given by
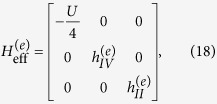
where
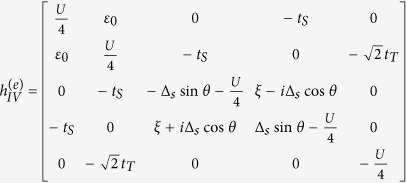
and
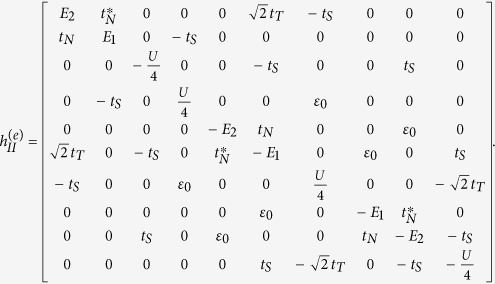


From these results, we can find that similar to the result in Case I, the different-FP matrix forms of *H*_eff_ obeys the relationship 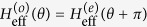.

## Diagonalization of *H* in Case III

In Case III, we turn to the discussion about the diagonalization of *H* when *t*_*T*_ ≪ *t*_*S*_. In such a case, the QD will dip in the *s*-wave superconductor, leading to the formation of a composite *s*-wave superconductor. Consequently, the considered structure will be transformed into a junction in which the Majorana doublet couples to a *s*-wave superconductor directly. Its Hamiltonian can thus be written as
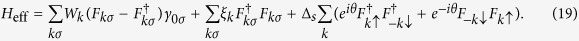


In Eq. (19), *F*_*kσ*_ originates from the unitary transformation that 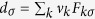 and 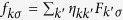, and *W*_*k*_ = *ν*_*k*_*t*_*T*_ denotes the coupling between the Majorana doublet and the *s*-wave superconductor. It is easy to prove that in composite *s*-wave superconductor couples weakly to the Majorana doublet [See the supplementary document]. As a result, the composite *s*-wave superconductor can be considered as perturbation. With respect to the Hamiltonian in Eq.(19), the action can be written as
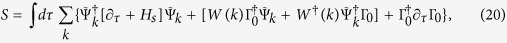
in which 
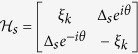
 and W(*k*) = *W*_*k*_*σ*_*z*_ with 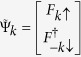 and 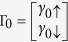. Similar to the derivation process in Case I, we express the partition function as a path integral
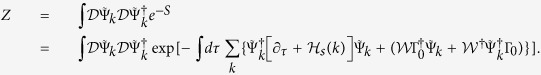


Integrating out the fermion field 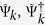 with a Gaussian integral, we simplify the partition function as
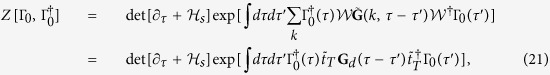
where 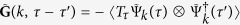 and 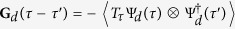 with 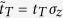.

Since **G**_*d*_(*τ*) obeys the relationship that 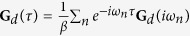, in the Fourier space the effective action can be expressed as 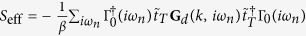. Accordingly, the Josephson Hamiltonian in Case III can be given by 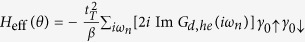. At the zero-frequency limit, the approximated form of *H*_eff_ can be written as *H*_eff_ = *J*(*iγ*_0↑_*γ*_0↓_)sin*θ* with
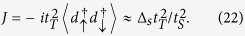


By defining 
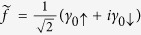
, we obtain the result that 
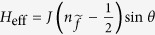
, i.e., 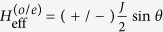. Therefore, the Josephson current can be directly written as 
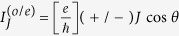
. Surely, such a result is consistent with that in ref. 32.

## Numerical Results and Discussions

Following the theory in the section above, we proceed to calculate the Josephson current in our considered junction. As a typical case, the system temperature is taken to be zero in the context. Besides, we take Δ_*s*_ = 1.0 to be the energy unit of the structural parameters.

**Figure 2:**
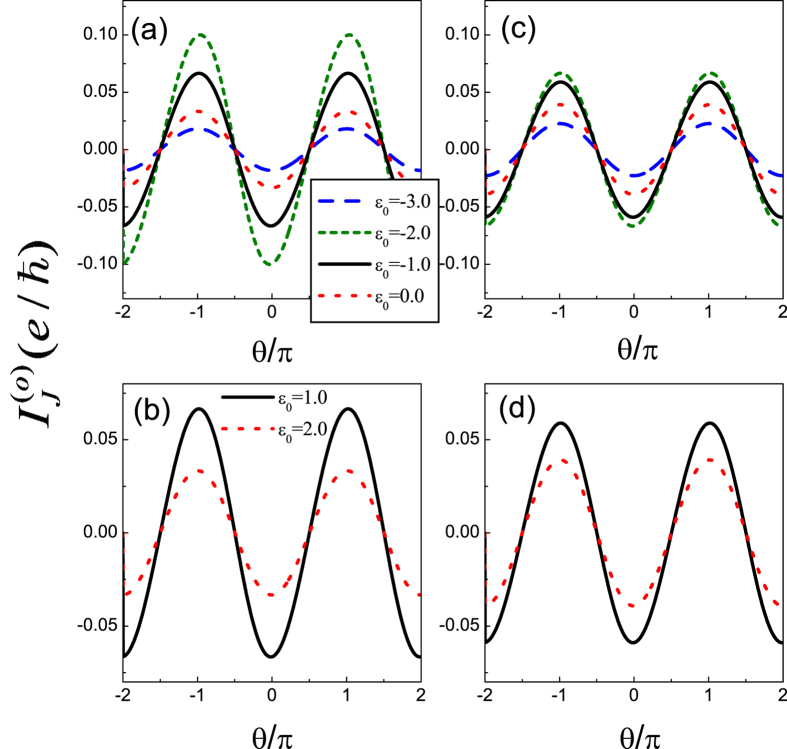
Spectra of odd-FP Josephson current in Case I of *U* = 0 and *U* = 2.0, respectively. The coupling strengths between the QD and superconductors are taken to be *t*_*T*_ = 0.5 and 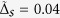. (**a,b**) *U* = 0; (**c,d**) *U* = 2.0.

First of all, we would like to investigate the odd-FP Josephson current in Case I. In Fig. 2, we plot the spectra of the Josephson current as a function of phase difference *θ*. As for the QD level, we change *ε*_0_ from −3.0 to 2.0. Besides, the intradot Coulomb strength is assumed to be 0.0 and 2.0, respectively. In this figure, we can find that despite the change of *ε*_0_ and *U*, the leading oscillation property of the Josephson current is relatively robust, since it reaches the maximum at the point *θ* = *nπ* with its profile as 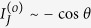. Meanwhile, the roles of QD level and Coulomb strength can be clearly observed. For instance, with the departure of the QD level from energy zero point, the current amplitude will be suppressed gradually. Such a result is relatively apparent in Fig. 2(a,b) which correspond to the case of the zero Coulomb interaction. The reason can be explained as the weakness of quantum coherence when the QD level departs from energy zero point. For the effect of Coulomb interaction, it is more apparent in the region of *ε*_0_ < 0, where the QD level is occupied. It can be seen that the Coulomb interaction suppresses the current amplitude as well. This should be attributed to the destruction of the quantum coherence induced by the QD-level splitting (*ε*_0_ → *ε*_0_ and *ε*_0_ + *U*) in the presence of Coulomb interaction.

**Figure 3:**
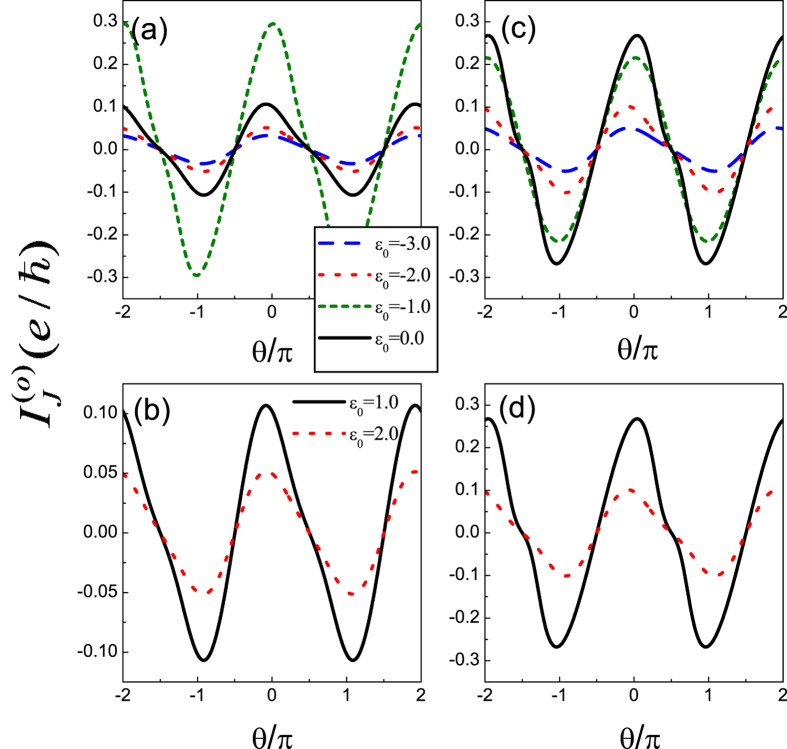
Odd-FP current in Case II of *U* = 0 and *U* = 2.0. In (**a,b**) *U* = 0, and *U* = 2.0 in (**c,d**). Relevant parameters are chosen as *t*_*T*_ = *t*_*S*_ = 0.5.

According to the discussion about Case II in the second part of Sec. II, the *s*-wave superconductor should not be viewed as perturbation when *t*_*S*_ gets close to *t*_*T*_. It is easy to think that in such a case, the Josephson current will show new properties. Thus, we would like to increase the coupling strength between the QD and *s*-wave superconductor to discuss the change of Josephson effect. The numerical results are shown in Fig. 3 where the *t*_*T*_ = *t*_*S*_ = 0.5. In this figure, we see that in Case II, the current properties are completely different from those in Case I. To be concrete, the current amplitude is efficiently enhanced and the current direction is completely reversed by the increase of *t*_*S*_. The other result is that the current profile deviates from the relationship of 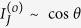 when *ε*_0_ ≠ 0. Such a phenomenon can be understood as follows. In the case of *t*_*T*_ = *t*_*S*_, practical Cooper-pair tunneling occurs between the TS and *s*-wave superconductor via the QD. When the QD level departs from the energy zero point, the phases of electron and hole are modified, and then the Cooper-pair tunneling process is changed.

**Figure 4:**
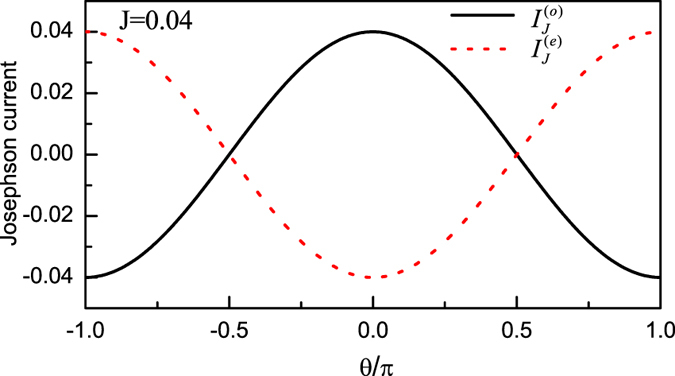
Josephson currents in Case III. The relevant parameters are taken to be *t*_*T*_ = 0.1 and *t*_*S*_ = 0.5. In the case of Δ_*s*_ = 1.0, the current amplitude *J* will be equal to be 0.04.

In what follows, if the coupling between the QD and *s*-wave superconductor further increases, the QD will be submerged in the *s*-wave superconductor. Consider the extreme case of the weak-coupling limit (i.e., Case III), the perturbation method can also be employed to evaluate the Josephson current, as displayed in the third part in Sec. II. It clearly shows that in such a case, Majorana doublet couples weakly to the composite *s*-wave superconductor. Consequently, the *s*-wave superconductor contributes an effective coupling between the two MBSs of one Majorana doublet. This exactly causes 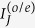 to follow the relationship that 
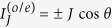
. In such case, the current properties become very well-defined, as described by the results in Fig. 4.

**Figure 5:**
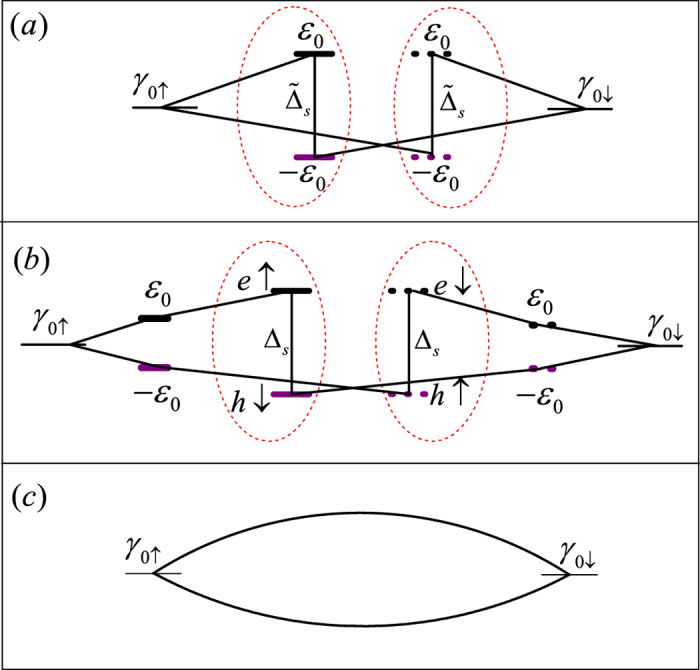
(**a,b**) Geometries of the Josephson junction in Case I, Case II, and Case III in the Nambu representation, respectively.

In view of the current results in Case I, Case II, and Case III, one can observe that at the case of *t*_*T*_ ≫ *t*_*S*_ (i.e., Case I), the current oscillation manner is opposite to that in the other two cases. In order to clarify the change of Josephson current from Case I to Case III, we plot the geometries of these three cases in the Nambu representation, as shown in Fig. 5. In Fig. 5(a–c), we notice that the finite coupling between the two MBSs of Majorana doublet, despite the direct or indirect coupling, gives rise to the occurrence of anomalous Josephson effect. On the other hand, the coupling strength between the QD and *s*-wave superconductor can vary the inter-MBS coupling property, leading to the change of the current oscillation manner. In the case of *t*_*T*_ ≪ *t*_*S*_, the MBSs couple directly to each other with a constant coupling parameter. In such a situation, the current direction is only dependent on the FP of the Majorana doublet with 
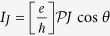
. In the other case where *t*_*T*_ ≫ *t*_*S*_, the coupling between the QD and Majorana doublet induces the indirect inter-MBS coupling, as shown in Fig. 5(a). With respect to the inter-MBS coupling in these two cases, we find that in the former case, the MBSs couple to each other via a nonresonant Andreev reflection process, whereas in the latter case, one bound state is involved in the Andreev reflection process. It is well known that the quasi-particle phase will undergo a *π*-phase shift due to the presence of one bound state in the Andreev reflection process. Accordingly, for the case of identical FP, the current oscillations in Case I and Case III are opposite to each other. By the same token, we can easily see that in Case I and Case II, the oscillation manners of the Josephson current are opposite to each other, because an additional bound state is presented in the Andreev reflection process in Case II. Up to now, we have known the reason that in the considered junction, the current in the case of *t*_*T*_ ≫ *t*_*S*_ is different from that in the other cases. Note, additionally, that in such a structure, the role of the *s*-wave superconductor is to provide a channel for the coupling between the MBSs in the Kramers doublet and the QD is to change the channel property. Due to this reason, the change of *ε*_0_ and *U* cannot induce any phase transition behaviors for the Josephson effect.

## Summary

To summarize, we have discussed the Josephson effects in the junction formed by the indirecting coupling between a one-dimensional *DIII*-class TS and a *s*-wave superconductor via an embedded QD. Via considering three QD-superconductor coupling manners, i.e., *t*_*T*_ ≫ *t*_*S*_, *t*_*T*_ = *t*_*s*_, and *t*_*T*_ ≪ *t*_*s*_, we have presented a comprehensive analysis about the Josephson effect in this system. As a consequence, it has been found that the Josephson current oscillates in period 2*π*. Moreover, the presence of Majorana doublet in the *DIII*-class TS renders the Josephson current finite in the case of zero phase difference between the superconductors. The other interesting result is that in addition to the FP of this system, the coupling strength between the QD and *s*-wave superconductor can affect the current direction. To be concrete, when the coupling between the QD and *s*-wave superconductor decreases to its weak limit, the direction of the Josephson current will have an opportunity to reverse. After analyzing the particle motion in this structure, we have demonstrated the reason for such a result. Namely, the QD-superconductor coupling manner can modulate the property of the Andreev reflection between the MBSs in the Majorana doublet. We believe that this work can be helpful for understanding the transport properties of the *DIII*-class TS.

At last, we notice that some previous work has also reported the nonzero-supercurrent phenomenon in the case of zero phase difference between two superconductors. For instance, ref. 37 describes a Josephson junction between two *s*-wave superconductors with an embedded QD. It shows that in the presence of spin-orbit interaction and a suitably oriented Zeeman field in the QD, the spontaneously-broken TRS leads to an anomalous supercurrent at zero phase difference between the superconductors. Therefore, this work is completely different from ours in the aspects of structure and result.

## Additional Information

**How to cite this article**: Gao, Z. *et al*. Josephson effects in the junction formed by *DIII*-class topological and *s*-wave superconductors with an embedded quantum dot. *Sci. Rep.* **6**, 28311; doi: 10.1038/srep28311 (2016).

## Supplementary Material

Supplementary Information
