# Supplementary material for: Josephson effects in the junction formed by DIII-class topological and s-wave superconductors with an embedded quantum dot
Source: Sci Rep. 2016 Jun 21;6:28311. doi: 10.1038/srep28311 (PMC4915010; doi:10.1038/srep28311)
Supplement: Supplementary Information [file srep28311-s1.pdf]

Supplementary material for Josephson effects in the junction formed by *DIII*-class topological and s-wave superconductors with an embedded quantum dot

Zhen Gao, Xiao-Qi Wang, Wan-Fei Shan, Hai-Na Wu, and Wei-Jiang Gong
College of Sciences, Northeastern University, Shenyang 110819, China

According to the Hamiltonian in Eq.(19) in the paper, the coupling strength between the Majorana doublet and the s-wave superconductor, i.e., $\tilde{\Gamma}_\sigma = \pi\sum_k |W_k|^2\rho_\sigma$, where ρ_σ is the density of state in the s-wave superconductor. Via a straightforward derivation, one can get the result that $\tilde{\Gamma}_\sigma = -t_T^2\text{Im}G^r_{d\sigma}$. $G^r_{d\sigma}$, defined by $G^r_{d\sigma}(t) = -i\theta(t)\langle\{d_\sigma(t), d^\dagger_\sigma\}\rangle$, is a retarded Green function of one QD coupled to a s-wave superconductor. By means of the nonequilibrium Green function technique, the matrix of the retarded Green function $\mathbf{G}^r_d$ can be obtained, i.e.,

$$[\mathbf{G}^r_d]^{-1} = \begin{bmatrix} (\omega-\varepsilon_0)S_{e\uparrow}+i\Gamma_0\rho_0 & 0 & 0 & -i\frac{\Delta_s}{\omega}\Gamma_0\rho_0 \\ 0 & (\omega-\varepsilon_0)S_{e\downarrow}+i\Gamma_0\rho_0 & -i\frac{\Delta_s}{\omega}\Gamma_0\rho_0 & 0 \\ 0 & -i\frac{\Delta_s}{\omega}\Gamma_0\rho_0 & (\omega+\varepsilon_0)S_{h\uparrow}+i\Gamma_0\rho_0 & 0 \\ -i\frac{\Delta_s}{\omega}\Gamma_0\rho_0 & 0 & 0 & (\omega+\varepsilon_0)S_{h\downarrow}+i\Gamma_0\rho_0 \end{bmatrix}, \quad (1)$$

where $\Gamma_0 = \pi\sum_k |V_{kS}|^2\delta(\omega-\xi_k)$ and $\rho_0 = \frac{|\omega|}{\sqrt{\omega^2-\Delta_s^2}}$. $S_{e(h)\sigma} = \frac{\omega\pm\varepsilon_0\pm U}{\omega\pm\varepsilon_0\pm U\mp U\langle n_{\bar{\sigma}}\rangle}$ is the effect of the electron interaction within the Hubbard-I approximation and $\langle n_{\bar{\sigma}}\rangle$ is the average electron occupation number. In the absence of magnetic factors, such a system is spin-degenerated, hence $\mathbf{G}^r_d$ can be simplified to be 2×2 matrix, i.e.,

$$\mathbf{G}^r_d = \begin{bmatrix} (\omega-\varepsilon_0)S_e+i\Gamma_0\rho_0 & -i\frac{\Delta_s}{\omega}\Gamma_0\rho_0 \\ -i\frac{\Delta_s}{\omega}\Gamma_0\rho_0 & (\omega+\varepsilon_0)S_h+i\Gamma_0\rho_0 \end{bmatrix}^{-1}. \quad (2)$$

In the limit of strong QD-superconductor coupling, the influence of ω and ε_0 will be submerged, and $G^r_{d\sigma}$ can be approximated as $-i\rho_0/\Gamma_0$, thus $\tilde{\Gamma}_\sigma \approx t_T^2\rho_0/\Gamma_0$. With the increment of t_S, Γ_0 will increase. This surely leads to the decrease of $\tilde{\Gamma}_\sigma$.
